# Comparison of ADMIRE, SAFIRE, and Filtered Back Projection in Standard and Low-Dose Non-Enhanced Head CT

**DOI:** 10.3390/diagnostics15121541

**Published:** 2025-06-17

**Authors:** Georg Gohla, Anja Örgel, Uwe Klose, Andreas Brendlin, Malte Niklas Bongers, Benjamin Bender, Deborah Staber, Ulrike Ernemann, Till-Karsten Hauser, Christer Ruff

**Affiliations:** 1 Department of Diagnostic and Interventional Neuroradiology, Eberhard Karls-University Tuebingen, D-72076 Tuebingen, Germanytill-karsten.hauser@med.uni-tuebingen.de (T.-K.H.); 2 MVZ Radiologie Karlsruhe GbR, Karlstraße 104-106, D-76137 Karlsruhe, Germany; 3 Department of Diagnostic and Interventional Radiology, Eberhard Karls-University Tuebingen, D-72076 Tuebingen, Germany

**Keywords:** image reconstruction, iterative reconstruction techniques, filtered back projection, SAFIRE, ADMIRE, dose reduction

## Abstract

**Background/Objectives**: Iterative reconstruction (IR) techniques were developed to address the shortcomings of filtered back projection (FBP), yet research comparing different types of IR is still missing. This work investigates how reducing radiation dose influences both image quality and noise profiles when using two iterative reconstruction techniques—Sinogram-Affirmed Iterative Reconstruction (SAFIRE) and Advanced Modeled Iterative Reconstruction (ADMIRE)—in comparison to filtered back projection (FBP) in non-enhanced head CT (NECT). **Methods**: In this retrospective single-center study, 21 consecutive patients underwent standard NECT on a 128-slice CT scanner. Raw data simulated dose reductions to 90% and 70% of the original dose via ReconCT software. For each dose level, images were reconstructed with FBP, SAFIRE 3, and ADMIRE 3. Image noise power spectra quantified objective image noise. Two blinded neuroradiologists scored overall image quality, image noise, image contrast, detail, and artifacts on a 10-point Likert scale in a consensus reading. Quantitative Hounsfield unit (HU) measurements were obtained in white and gray matter regions. Statistical analyses included the Wilcoxon signed-rank test, mixed-effects modeling, ANOVA, and post hoc pairwise comparisons with Bonferroni correction. **Results**: Both iterative reconstructions significantly reduced image noise compared to FBP across all dose levels (*p* < 0.001). ADMIRE exhibited superior image noise suppression at low (<0.51 1/mm) and high (>1.31 1/mm) spatial frequencies, whereas SAFIRE performed better in the mid-frequency range (0.51–1.31 1/mm). Subjective scores for overall quality, image noise, image contrast, and detail were higher for ADMIRE and SAFIRE versus FBP at the original dose and simulated doses of 90% and 70% (all *p* < 0.001). ADMIRE outperformed SAFIRE in artifact reduction (*p* < 0.001), while SAFIRE achieved slightly higher image contrast scores (*p* < 0.001). Objective HU values remained stable across reconstruction methods, although SAFIRE yielded marginally higher gray and white matter (WM) attenuations (*p* < 0.01). **Conclusions**: Both IR techniques—ADMIRE and SAFIRE—achieved substantial noise reduction and improved image quality relative to FBP in non-enhanced head CT at standard and reduced dose levels on the specific CT system and reconstruction strength tested. ADMIRE showed enhanced suppression of low- and high-frequency image noise and fewer artifacts, while SAFIRE preserved image contrast and reduced mid-frequency noise. These findings support the potential of iterative reconstruction to optimize radiation dose in NECT protocols in line with the ALARA principle, although broader validation in multi-vendor, multi-center settings is warranted.

## 1. Introduction

The availability and the annual frequency of computed tomography (CT) examinations have increased dramatically in recent decades [1]. Throughout the years, patients and operators have become increasingly critical about radiation exposure and the risk for its long-term effects [2,3]. As radiation risk increases with repeated CT examinations, for example, for the emergency assessment of patients with acute-onset neurological deficits or after recent traumatic injuries, we need reliable instruments to minimize the radiation dose exposure with every non-enhanced head CT (NECT). While considering radiation risk, high image quality should be maintained for high diagnostic accuracy [4,5,6]. Accordingly, reduced-dose NECT protocols should aim to optimize the trade-off between diagnostic image quality and radiation exposure, in alignment with the ALARA (as low as reasonably achievable) principle. Low-dose NECT techniques can provide diagnostically suitable images while reducing the patient radiation dose [7,8,9].

So far, numerous techniques, including peak voltage optimization, tube current modulation, improved detection system efficacy, and iterative image reconstruction (IR) techniques, have been continuously developed to reduce radiation dose [10,11,12,13,14]. However, the ionizing radiation dose should not be reduced arbitrarily, as this leads to non-diagnostic NECT examinations due to the inversely proportional image noise to the square root of the tube current [15]. In particular, subtle low-image contrast differences and anatomical structures can suffer from the reduced contrast-to-noise ratio (CNR) [16,17].

For many years, filtered back projection (FBP) has served as the conventional technique for image reconstruction in computed tomography. However, it is limited by its inability to effectively suppress image noise or correct artifacts, leading to grainy images and an impaired visualization of subtle anatomical structures, especially in low-dose protocols [18]. These limitations compromise diagnostic accuracy, particularly in neuroradiological applications, where fine detail is crucial.

To address these shortcomings, IR techniques were developed. By repeatedly refining image data using mathematical models, IR methods achieve improved image noise suppression and higher spatial resolution than FBP [19]. Although they can prolong reconstruction time and can produce images with an unnatural appearance at high-strength settings and low-dose acquisitions, IR techniques represent a significant advancement in CT imaging [20]. IR has many advantages over FBP, especially with neuroradiological CT exams [6,10,11,21,22]. To our knowledge, only a few studies directly compared the different IR algorithms like sinogram-affirmed IR (SAFIRE, Siemens Healthineers, Erlangen, Germany) and advanced modeled IR (ADMIRE, Siemens Healthineers, Erlangen, Germany) in neuroradiology exams in CT angiography of supra-aortic arteries, circle of Willis, or CT of the head and neck [12,23,24,25]. However, there is a lack of low-dose studies investigating the diagnostic accuracy of different IR algorithms.

This study aimed to examine the SAFIRE and ADMIRE IR algorithms at different dose levels. In light of this, the present study explores the impact of radiation dose reduction on the diagnostic accuracy, image noise levels, and image quality of the SAFIRE and ADMIRE (each at strength level 3) IR techniques compared to conventional FBP on NECT.

## 2. Materials and Methods

### 2.1. Study Design

**Figure 1:**
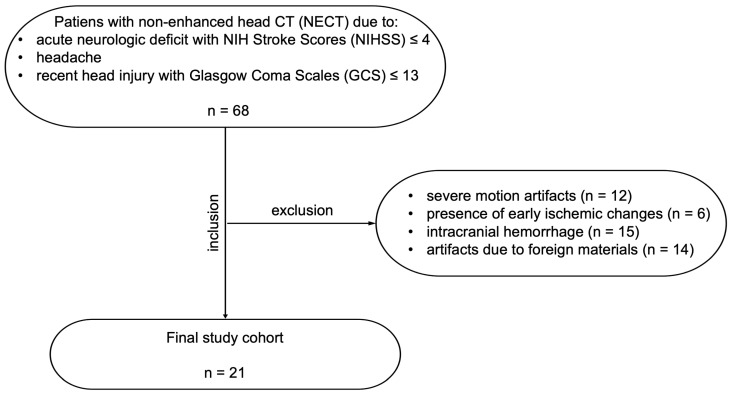
Study flow chart of patient inclusion and exclusion criteria.

This retrospective single-center study received approval from the local ethics committee (232/2019B02), with a waiver of informed consent. All procedures complied with the principles set forth in the Declaration of Helsinki. A priori power analysis was performed using G*Power (version 3.1.9.7) to determine the required sample size for Hounsfield unit (HU) comparisons. Assuming an alpha level of 0.05, a statistical power of 0.859, and an expected effect size value of 2.329, a total sample size of *n* = 4 per group was calculated. These parameters were based on the previous literature [26]. In our clinical routine, we included 68 consecutive patients with clinically indicated NECT from one scanner. The inclusion and exclusion criteria are detailed in Figure 1. We collected the patient’s sex and age at the examination time. We extracted the CT dose parameters from the DICOM metadata, including the volume CT dose index (CTDIvol, in mGy), dose-length product (DLP, in mGy·cm), and effective tube current (in mAs). To estimate the effective dose (in mSv), the DLP was multiplied by the ICRP-recommended conversion coefficient for head CT (0.0021 mSv/mGy·cm) [27].

### 2.2. Scanning Protocol and Reconstruction Parameters

All patients were examined on a 128-slice CT scanner with stellar detectors (Somatom Definition AS; Siemens Healthineers, Erlangen, Germany) and the software version syngo CT VA48A with image acquisition parameters of the standard protocol (for the scanning parameters see Table 1). A fixed tube voltage of 120 kV was used, while the tube current was individually adapted based on head size through automatic attenuation-based modulation (CAREDose4D, Siemens Healthineers, Erlangen, Germany; reference value: 350 mAs). The field of view was set to 220 × 220 mm. The image quality of the NECT protocol has been evaluated and confirmed in an earlier studies [28].

SAFIRE and ADMIRE cannot be used simultaneously for CT reconstruction on one device. To simulate examinations with reduced tube current and consequently lower radiation dose, the ReconCT software (Siemens Healthineers, Erlangen, Germany) was employed to generate realistic low-dose CT datasets from standard high-dose scans. In detail, raw data were anonymized and transferred to a dedicated offline workstation with the prototype software ReconCT (version 14.2.0.40998, Siemens Healthineers, Erlangen, Germany) for the reconstruction of conventional FBP, SAFIRE, and ADMIRE images from the same raw data. Previous studies have proven the validity of this software in CT angiography of supra-aortic arteries [23,29], neck CT [30], or body CT [31,32,33].

Reconstruction of the scans was performed in the axial plane using a medium smooth convolution kernel (H30s) for FBP and a corresponding medium smooth kernel (J30) for iterative reconstruction. Images were reformatted along the anterior commissure–posterior commissure (AC-PC) line with a slice thickness and increment of 4 mm. For IR, we used sinogram-affirmed iterative reconstruction (SAFIRE strength level 3, Siemens Healthineers, Erlangen, Germany) and advanced modeled iterative reconstruction (ADMIRE strength level 3, Siemens Healthineers, Erlangen, Germany). Furthermore, realistic low-dose images at 90% and 70% of the initial dose were reconstructed using the same three reconstruction algorithms, FBP, SAFIRE (strength level 3), and ADMIRE (strength level 3). The matrix size of the reconstructed images was 512 × 512, with a pixel size of 0.429 × 0.429 mm^2^.

### 2.3. Objective Assessment of Image Noise

The image noise intensity in reconstructed images was evaluated by image noise power spectra (NPS). All reconstructions were performed five times with identical parameters to obtain image noise images from reconstructed data. A mean image was calculated and subtracted from the five reconstruction images. A power map was obtained by averaging the five difference images and evaluating the absolute values of the complex values after a two-dimensional Fourier transformation. Next, directional power spectra were calculated by selecting values of the power map along 18 straight lines with different angles (10° to 180° in steps of 10°) between the horizontal and the vertical axis. An average of all 18 directional power spectra gave us a power spectrum that no longer depended on a chosen angle. For each slice depicting parts of the hemispheres or the cerebellum, the power spectrum was calculated and averaged into a final spectrum that resembled an average of the whole cerebrum. This power spectrum was used to compare the image noise content of images reconstructed with different reconstruction parameters.

### 2.4. Subjective Image Quality

Two neuroradiologists with 20 (reader 1) and 6 (reader 2) years of experience in neuroradiologic imaging and who were not aware of the reconstruction information assessed the 21 image datasets in a random order. The evaluated datasets consisted of the FBP, SAFIRE, and ADMIRE reconstruction images at the original dose and simulated doses only at 90% and 70% of the original dose, as a publication by Korn et al. showed that a 30% dose reduction results in a substantial increase in noise despite using iterative reconstruction [28]. Both reviewers were blinded to the reconstruction method and to the clinical and radiological findings, as well as to each other’s assessments. Identifying patient information and sequence labels were removed, and the images were displayed without any annotations. The evaluations were performed using a dedicated workstation (syngo.via; Siemens Healthineers, Erlangen, Germany). These were performed in certified reading room conditions on certified diagnostic radiology monitors (RadiForce RX350, Eizo Corporation, Hakusan, Ishikawa, Japan). The image datasets were evaluated in a consensus reading of both readers together for overall image quality, subjective image noise, image detail, subjective image contrast, and artifacts by using a 10-point Likert scale, with a score of 10 representing the best outcome (1  =  very poor; 10 = very good).

### 2.5. Objective Image Quality

For objective image analysis, regions of interest (ROIs) were placed in consensus readings in identical supratentorial frontal periventricular white matter (WM) and gray matter (GM, thalamus) locations in a mid-thalamic slice (Supplementary Figure S1). This placement was applied for each patient and for each FBP and IR image. The image processing program OsiriX MD (Pixmeo SARL, Geneva, Switzerland) was utilized for this analysis. Mean ROI density (Hounsfield units) was evaluated.

### 2.6. Statistical Analysis

Statistical analyses were performed with SPSS Statistics software (SPSS, Version 30, IBM Corp., Armonk, NY, USA). Image noise analysis was performed with MATLAB R2015b (The MathWorks, Inc., Natick, MA, USA). Quantitative data were shown as mean ± standard deviation (mean ± SD), and relative frequencies were expressed as *n* (%). Prior to conducting 1-way analysis of variance (ANOVA) on HU data between reconstruction algorithms, we formally tested the assumptions of normality and homoscedasticity. Normality was assessed using the Lilliefors-corrected Kolmogorov–Smirnov test, which adjusts for the estimation of population parameters. Given our moderate sample size (*n* = 21 per group), we applied a conservative significance threshold of *p* ≥ 0.01. Since the D-statistic scales with sample size, we based the interpretation on the *p*-value rather than the raw D. Homoscedasticity was tested using Levene’s test. As both assumptions were met across all groups, classical one-way ANOVA was deemed appropriate and applied throughout. Data were analyzed using a mixed-effects model, applying the Greenhouse–Geisser correction when the sphericity assumption was violated. To adjust for multiple comparisons and reduce the risk of type I errors, Bonferroni correction was also employed. Regarding objective imaging parameters, intra-dose comparisons between ADMIRE and SAFIRE were performed by paired *t*-tests. Subjective image quality comparisons between original dose and dose simulations utilized the Wilcoxon signed-rank test. An adjusted *p*-value ≤ 0.05 indicated statistical significance.

## 3. Results

### 3.1. Study Population and Radiation Dose

This retrospective study included 21 consecutive patients with a mean age of 63 ± 11 years (9 males, 12 females; age range: 19 to 90 years). The CTDIvol and DLP of our standard NECT protocol were 41.9 ± 2.9 mGy (range 36.48–47.01 mGy) and 638.4 ± 65.4 mGycm (range 534.6–718.0 mGycm). The effective dose was 1.34 ± 0.14 mSv (range 0.74–5.89 mSv). The mean effective tube current was 401.6 ± 28.7 mAs.

### 3.2. Image Noise

**Figure 2:**
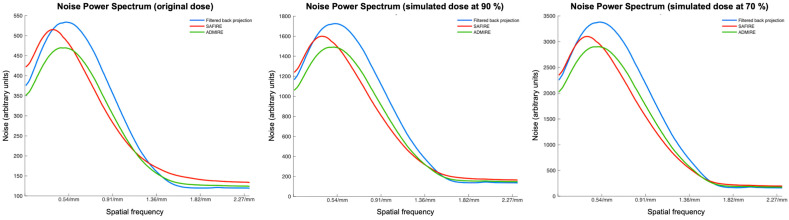
Objective image noise quantification by image noise power spectra (NPS). The frequency distribution of image noise (expressed by NPS) indicates that the image noise is higher in filtered back projection (FBP) reconstruction at the original dose and at simulated dose reductions to 90% and 70%. Iterative reconstruction algorithms shift the image NPS toward lower spatial frequencies at all dose reconstructions, which is more pronounced for ADMIRE than for SAFIRE. NPS = noise power spectra; FBP = filtered back projection; ADMIRE = advanced modeled iterative reconstruction; SAFIRE = sinogram-affirmed iterative reconstruction.

The frequency distribution of image noise (expressed by NPS) indicated that the image noise was significantly higher in FBP reconstruction for the original dose and the simulated doses at 90% and 70% compared to iterative reconstruction methods (Figure 2, for all *p* < 0.001).

**Figure 3:**
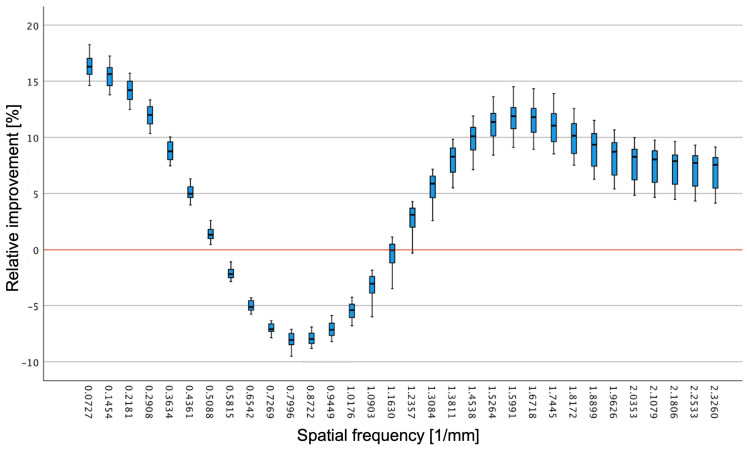
Relative improvement of ADMIRE in comparison to SAFIRE reconstruction. ADMIRE showed improved noise levels at low spatial frequency (≤0.5088/mm), whereas SAFIRE showed lower noise levels at spatial frequency between 0.5088/mm and 1.3084/mm. The image marks this by the red line at 0 (relative improvement in [%]) with values above or below it depending on the difference between ADMIRE and SAFIRE. ADMIRE = advanced modeled iterative reconstruction; SAFIRE = sinogram-affirmed iterative reconstruction.

The direct comparison between SAFIRE and ADMIRE revealed that ADMIRE showed superior image noise suppression at lower spatial frequencies below 0.5088 1/mm and higher spatial frequencies above 1.3084 1/mm in a comparison across all dose simulations. In comparison, SAFIRE showed lower image noise between 0.5088 1/mm and 1.3084 1/mm (Figure 3, for all *p* < 0.001).

### 3.3. Subjective Image Quality Analysis

**Figure 4:**
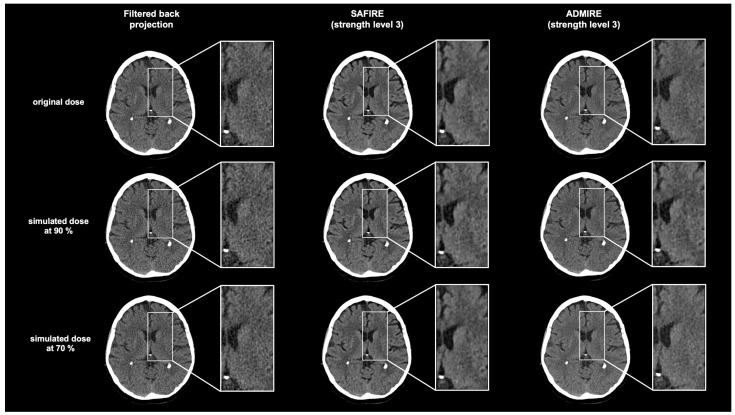
Head-to-head comparison of example images at original dose and ReconCT dose simulations of 90% and 70% in filtered back projection (FBP), in sinogram-affirmed iterative reconstruction (SAFIRE), and in advanced modeled iterative reconstruction (ADMIRE) of a non-enhanced head computed tomography (NECT). At each dose setting, the gray matter of the basal ganglia and the thalamus, as well as the frontal periventricular white matter, are shown enlarged as zoom-in boxes. FBP = filtered back projection; ADMIRE = advanced modeled iterative reconstruction; SAFIRE = sinogram-affirmed iterative reconstruction.

Figure 4 shows a head-to-head comparison of example images at the original dose and ReconCT dose simulations of 90% and 70% in FBP, SAFIRE, and ADMIRE. Detailed scoring results are shown in Table 2.

The results between the dose gradations within the specific reconstruction algorithms are mostly significant. Exceptions include some quality metrics between the original dose and the simulated dose of 90%, such as the image noise for FBP, contrast and image detail for SAFIRE, and overall image quality and contrast and artifacts for ADMIRE. Mixed-effects analysis of the pooled subjective quality scores showed significant interactions between the semiquantitative score points of the datasets (F (8.160) = 1000.731; *p* < 0.001; np² = 0.980).

In the corrected pairwise post hoc comparisons, both iterative techniques received higher scores than FBP at the original dose and radiation dose simulations of 90% and 70% in the categories of image quality, image detail, image contrast, and image noise (each *p* < 0.001). For image quality, ADMIRE showed slightly higher scores than SAFIRE at all radiation doses (each *p* < 0.001). However, a dose reduction from the original dose to 90% demonstrated no significant difference in image quality (*p* ≥ 0.958), and further dose reduction to 70% was accompanied by a minimally significant decrease in image quality (each *p* < 0.001). For image detail and noise, both iterative techniques revealed higher scores than FBP at all radiation dose simulations (each *p* < 0.001). However, ADMIRE received higher scores than SAFIRE and FBP at all radiation dose simulations in image detail (each *p* < 0.001). For image contrast, both iterative techniques revealed higher scores than FBP at all radiation doses (each *p* < 0.001). In comparison, SAFIRE scored higher than ADMIRE and FBP at all radiation doses (each *p* < 0.001). In the artifact category, ADMIRE showed fewer artifacts compared to SAFIRE and FBP for all doses (each *p* < 0.001), while SAFIRE demonstrated more artifacts than FBP for all doses (each *p* < 0.001). Supplementary Figure S2 shows an example image for artifacts using SAFIRE (strength level 3) compared to FBP and ADMIRE.

### 3.4. Objective Image Quality Analysis

Table 3 presents the findings from the objective image quality analysis of white and gray matter density for the original dose and simulated dose at 90% and 70% for FBP and for SAFIRE and ADMIRE reconstructions. The original dose and the dose-reduced reconstructions result in significant changes in the measured HU values for gray matter between the reconstruction types.

SAFIRE reconstructions showed significantly higher HU values for GM at the original dose and at simulated doses of 90% and 70% compared to FBP and ADMIRE (Table 4, for all *p* ≤ 0.002). All other comparisons, especially between the corresponding original dose and a 90% dose level, revealed no significant differences (Table 4, for all *p* > 0.180).

## 4. Discussion

NECT is an indispensable imaging technique for a wide range of neurological and neurosurgical questions in the diagnosis of acute disorders and the follow-up of various diseases. It plays a pivotal role in therapeutic decision-making. Over time, concerns about radiation exposure have grown among both patients and healthcare providers, particularly given the rising use of NECT in emergency and inpatient settings, along with the potential long-term carcinogenic risks, especially in younger patients. To date, evidence emphasizes the careful justification of CT imaging and the use of doses as low as reasonably possible.

Iterative reconstruction methods have demonstrated the potential to lower radiation doses by 20% to 40% in NECT while also enhancing the detection of low-contrast structures compared to conventional FBP [6,10,11,21,22,25,34]. Therefore, this study aimed to assess the impact of ADMIRE and SAFIRE at different dose levels. Our hypothesis was that the investigated algorithms would allow for maintaining high-quality NECT images at reduced radiation doses compared to traditional FBP.

The investigation of image noise demonstrated that both iterative reconstruction types (ADMIRE and SAFIRE) produce less image noise than FBP. In image contrast, the differences within both iterative methods depend on the spatial frequencies. The image noise performance of the ADMIRE reconstruction type is superior to that of SAFIRE at low and high spatial frequencies. At the same time, SAFIRE presents lower image noise than ADMIRE at spatial frequencies in the medium range between 0.5088 and 1.3084.

Our findings are in close agreement with prior work demonstrating that iterative reconstruction algorithms induce a shift in the image noise power spectrum (NPS) toward lower spatial frequencies, yielding a “blotchy” texture that is more pronounced for ADMIRE than for SAFIRE. Dalehaug et al. compared ADMIRE and SAFIRE in phantom experiments and showed that, at reduced dose levels, the median of the NPS curves for both algorithms shifts toward lower frequencies, with ADMIRE exhibiting a larger shift at all strength settings [35]. Similarly, ensemble-averaging measurements by Yu et al. confirmed that SAFIRE’s image noise distribution degrades spatial resolution at very low image contrast levels, reflecting its nonlinear regularization [36].

In image contrast, ADMIRE offers superior image noise suppression at high spatial frequencies (>1.0 lp/mm), which translates into a reduction in the coefficient of variation (COV) and an enhancement of image contrast-to-image noise ratio (CNR). Raslau et al. evaluated ADMIRE strength levels 1–5 in a live ovine head CT model. They reported that ADMIRE strength level 3 achieved up to a 50% dose reduction at 120 kV while preserving gray-white differentiation and image texture, with marked improvements in low-image contrast detectability metrics [37].

Within the intermediate spatial frequency band (0.5088–1.3084 lp/mm), our observation that SAFIRE outperforms ADMIRE in image noise reduction is supported by Korn et al., who demonstrated in adult head CT that SAFIRE significantly improves signal-to-image noise (SNR) and CNR compared to FBP due to more homogeneous image noise suppression in soft-tissue regions [38]. This balance between image noise reduction and edge preservation may account for SAFIRE’s relative advantage in the mid-frequency range.

The subjective image quality assessment indicated that both iterative reconstruction methods, SAFIRE and ADMIRE, are superior to FBP in overall image quality, image detail, image noise, and image contrast. Additionally, both iterative reconstruction techniques demonstrated better subjective image quality in low-dose simulations than FBP at the original dose. These findings support those reported by Korn et al., who found that iterative reconstruction in head CT significantly enhances subjective image quality and permits dose reductions of up to 20% without sacrificing diagnostic integrity [28]. However, they did not differentiate between the two iterative techniques, SAFIRE and ADMIRE, and applied different tube currents. As a result, they emphasized the need for additional studies to enable more precise intra-patient comparisons. Rabinowich et al. investigated a varied pediatric cohort using low-dose NECT, including both normal and pathological scans [39]. Despite the diversity within their patient cohort, they observed that iterative model reconstructions provided superior image quality compared to FBP. These results are consistent with ours, even if we have evaluated the subjective image quality in a more differentiated way and have a different patient population. In line with our study, Rivers-Bowerman et al. showed that SAFIRE improves subjective image quality in adult NECT [38]. We examined the subjective image quality of two different iterative reconstruction techniques. In image contrast, our images reconstructed with SAFIRE demonstrated more or more impairing artifacts compared to ADMIRE. Several studies support our findings because of the higher CNR in ADMIRE than SAFIRE, and ADMIRE removed image noise near edges more efficiently, but no study evaluated both iterative techniques in NECT [35,40].

In objective image quality analysis, the mean HUs of WM and GM were unaffected by FBP and ADMIRE, while SAFIRE showed higher values for all dose simulations. Maintaining consistent CT numbers is crucial for accurately identifying and differentiating various brain tissues, anatomical structures, and pathological conditions. This necessity is particularly relevant given the inherent limitations of certain denoising approaches, which may lead to loss of information and reduced spatial resolution due to increased image blurring [41,42,43].

These findings may have direct clinical relevance. The preservation of HU accuracy in white and gray matter across dose levels—particularly with ADMIRE—supports reliable tissue differentiation, which is crucial in detecting subtle parenchymal changes such as early infarction, edema, or mass effect [25]. Additionally, improved image quality and artifact suppression may aid diagnostic confidence in anatomically challenging areas such as the posterior fossa [39]. Thus, the application of iterative reconstruction in routine NECT protocols offers a practical means to lower radiation dose without compromising diagnostic performance.

Several limitations of this study should be acknowledged. Firstly, for NPS calculation, the images are usually acquired several times to record image noise accurately. As this is impossible when using in vivo imaging, a software tool that can simulate image noise corresponding to different levels of dose reduction was used. Other studies have shown the reliability of ReconCT [12,23,29,30,31,32,33]. Additionally, ReconCT only allowed for axial reconstruction, whereas images are angled to the Talairach–Tournoux Line in the clinical setting. Secondly, the study utilized ReconCT to generate datasets representing 10% and 30% dose reduction, respectively. Although the platform’s Poisson-based noise insertion has been well validated for conventional image quality metrics, it cannot reproduce every physical component of genuine low-mAs acquisitions. The most notable of these are detector electronic read-out noise, tube current modulation-induced view angle dependencies, and energy-dependent beam-hardening effects. These factors have the potential to modify the noise texture, thereby restricting the direct applicability of our findings to low-dose images acquired prospectively. Thirdly, only the IR technology of a single CT manufacturer was used; therefore, the results cannot simply be transferred to other CT devices, as the technical approaches to IR differ between manufacturers. Fourthly, SAFIRE and ADMIRE allow different strength levels. Different strength settings may yield varying degrees of image noise suppression, image contrast preservation, and artifact expression. We selected SAFIRE and ADMIRE at strength level 3 exclusively, as these settings are routinely employed at our institution and offer a balanced compromise between noise reduction and preservation of the images’ visual characteristics. Fifthly, we did not correlate imaging findings with clinical outcomes or diagnostic performance metrics such as lesion detectability or reader diagnostic accuracy, which is essential to translate image-quality improvements into patient benefit. Finally, the use of consensus reading for both subjective and objective image assessment without reporting inter-reader agreement metrics may be seen as a methodological limitation. Nevertheless, this approach is not without merit. It reflects common clinical practice and has been adopted in several imaging studies [44,45,46,47], demonstrating that consensus-based ratings are both widely accepted and methodologically sound. Moreover, consensus reading helps to reduce random error, mitigate individual-reader bias, and establish a more stable reference standard. Future studies may nonetheless benefit from including individual reader ratings to further assess robustness across observers.

## 5. Conclusions

This investigation demonstrates that the SAFIRE and ADMIRE (strength level 3) iterative reconstruction algorithms outperform conventional FBP in non-enhanced head CT at standard and reduced radiation dose levels using the tested scanner platform. Iterative reconstruction lowers image noise across spatial frequencies and enhances subjective measures of image quality, detail, and image contrast. ADMIRE’s advanced modeling provides superior suppression of high- and low-frequency image noise. It minimizes artifacts, while SAFIRE achieves optimal image noise reduction in medium spatial frequencies and slightly higher tissue image contrast. Importantly, both IR methods sustain consistent Hounsfield unit accuracy across white and gray matter regions, underscoring their reliability for diagnostic interpretation. By enabling up to 30% dose reduction without compromising—or even improving—diagnostic image quality, these techniques align with the ALARA principle and potentially decrease patient radiation exposure in routine neuroradiologic practice. Future prospective studies with larger, multicenter cohorts and clinical outcome correlations are warranted to validate these results and facilitate widespread adoption across different CT platforms.

## Figures and Tables

**Table 1:** CT scanning protocol on a 128-slice CT scanner with stellar detectors (Somatom Definition AS; Siemens Healthineers, Erlangen, Germany) and the software version syngo CT VA48A for subsequent use of ReconCT software (version 14.2.0.40998, Siemens Healthineers, Erlangen, Germany).

Scanning Parameters	
scan mode	spiral
stellar detector configuration	128 × 0.6 (64 × 0.6 = 38.4 mm)
slice collimation (mm)	40 × 0.6
effective reference tube voltage (kV)	100
effective tube current-time product (mAs)	401.6 ± 28.7 (350–445)
pitch	0.55
rotation time (s)	1

mm = millimeter; kV = kilovolt; mAs = milliampere-second; s = second.

**Table 2:** Qualitative image quality for filtered back projection and the iterative reconstruction methods SAFIRE and ADMIRE using a Likert scale of 1–10, with 10 being the best score. The *p*-values were calculated using the Wilcoxon signed-rank test.

Category	Reconstruction Method			Radiation Dose Simulation				*p*-Value Original vs. 90%	*p*-Value Original vs. 70%	*p*-Value 90% vs. 70%
		Original		90%		70%				
		Mdn (IQR)	Mean ± SD	Mdn (IQR)	Mean ± SD	Mdn (IQR)	Mean ± SD			
Overall image quality	FBP	4 (4–5)	4.48 ± 0.51	4 (4–4)	3.95 ± 0.25	3 (2–3)	2.71 ± 0.46	0.001	<0.001	<0.001
	SAFIRE	8 (7–8)	7.57 ± 0.51	8 (8–8)	7.95 ± 0.21	7 (7–7)	6.95 ± 0.20	0.005	0.001	<0.001
	ADMIRE	9 (9–9)	8.95 ± 0.22	9 (9–9)	8.95 ± 0.22	8 (8–8)	7.95 ± 0.19	1	< 0.001	<0.001
Image detail	FBP	4 (3–4)	3.57 ± 0.51	3 (3–3)	2.86 ± 0.36	2 (2–2.5)	2.24 ± 0.44	0.001	<0.001	0.001
	SAFIRE	7 (7–7)	6.95 ± 0.21	7 (7–7)	7.05 ± 0.22	6 (6–6)	5.95 ± 0.23	0.157	<0.001	<0.001
	ADMIRE	9 (9–9)	8.95 ± 0.18	8 (8–9)	8.48 ± 0.51	7 (7–7)	6.95 ± 0.22	0.002	<0.001	<0.001
Image noise	FBP	4 (4–4)	3.95 ± 0.22	4 (4–4)	3.95 ± 0.23	3 (3–3)	2.95 ± 0.22	1	<0.001	<0.001
	SAFIRE	9 (8–9)	8.67 ± 0.48	8 (7.5–8)	7.76 ± 0.44	7 (7–7)	6.95 ± 0.22	<0.001	<0.001	<0.001
	ADMIRE	9 (8.5–9)	8.76 ± 0.44	8 (8–8)	8.05 ± 0.22	6 (6–6)	6.00 ± 0.45	<0.001	<0.001	<0.001
Contrast	FBP	5 (5–5)	4.95 ± 0.24	4 (4–5)	4.48 ± 0.51	3 (3–3)	2.95 ± 0.24	0.004	<0.001	<0.001
	SAFIRE	9 (9–9)	9.05 ± 0.22	9 (9–9)	8.95 ± 0.22	8 (8–8)	7.95 ± 0.24	0.157	<0.001	<0.001
	ADMIRE	7 (7–7)	6.95 ± 0.24	7 (7–7)	6.95 ± 0.22	6 (6–6)	5.95 ± 0.22	1	<0.001	<0.001
Artifacts	FBP	7 (7–7)	6.95 ± 0.22	6 (5–6)	5.48 ± 0.93	5 (5–5)	4.95 ± 0.21	<0.001	<0.001	0.036
	SAFIRE	5 (5–6)	5.48 ± 0.51	4 (4–4)	4.19 ± 0.40	4 (4–4)	3.95 ± 0.22	<0.001	<0.001	0.059
	ADMIRE	9 (9–9)	8.95 ± 0.22	9 (9–9)	8.95 ± 0.21	8 (8–8)	7.95 ± 0.24	1	<0.001	<0.001

FBP = filtered back projection, SAFIRE = sinogram-affirmed iterative reconstruction; ADMIRE = advanced modeled iterative reconstruction; Mdn = median; IQR = interquartile range; SD = standard deviation.

**Table 3:** Evaluation of objective image quality of white and gray matter density for the original dose and simulated dose at 90% and 70%. ROIs were placed in identical frontal periventricular white matter and gray matter areas in the thalamus. The *p*-values were calculated using ANOVA.

ROI Localization	Reconstruction Algorithm	Dose Simulation	Hounsfield Units (Mean ± SD)	*p*-Value
GM	FBP	original	39.916 ± 1.275	0.001
	SAFIRE	original	41.254 ± 1.251	
	ADMIRE	original	39.967 ± 1.180	
WM	FBP	original	33.452 ± 1.424	0.347
	SAFIRE	original	32.913 ± 1.703	
	ADMIRE	original	33.542 ± 1.472	
GM	FBP	90%	39.795 ± 1.280	0.001
	SAFIRE	90%	41.182 ± 1.335	
	ADMIRE	90%	39.870 ± 1.197	
WM	FBP	90%	33.339 ± 1.421	0.392
	SAFIRE	90%	32.847 ± 1.673	
	ADMIRE	90%	33.444 ± 1.494	
GM	FBP	70%	39.815 ± 1.246	< 0.001
	SAFIRE	70%	41.176 ± 1.317	
	ADMIRE	70%	39.843 ± 1.153	
WM	FBP	70%	33.325 ± 1.384	0.378
	SAFIRE	70%	32.843 ± 1.626	
	ADMIRE	70%	33.417 ± 1.490	

ROI = region of interest; SD = standard deviation; GM = gray matter; WM = white matter; FBP = filtered back projection; SAFIRE = sinogram-affirmed iterative reconstruction; ADMIRE = advanced modeled iterative reconstruction.

**Table 4:** ANOVA test with Bonferroni correction of difference between regions of interest (ROIs) in identical supratentorial white matter (frontal periventricular white matter) and gray matter locations (thalamus) partially showed significant changes in Hounsfield units at the original dose and simulated dose at 90% and 70% between FBP, SAFIRE, and ADMIRE reconstructions. Standard error of the mean (SEM) is given for the respective groups.

ROI Localization	Algorithm Group 1 vs. Group 2		Dose Simulation	Hounsfield Units (Mean Difference)	95% Confidence Interval	*p*-Value	SEM
GM	FBP	SAFIRE	original	−1.338	−2.212 to −0.460	0.001	0.356
		ADMIRE	original	−0.051	−0.926 to 0.823	1	
	SAFIRE	FBP	original	1.338	0.463 to 2.212	0.001	
		ADMIRE	original	1.286	0.412 to 2.161	0.002	
	ADMIRE	FBP	original	0.051	−0.823 to 0.926	1	
		SAFIRE	original	−1.286	−2.161 to −0.412	0.002	
WM	FBP	SAFIRE	original	0.539	−0.550 to 1.627	0.685	0.443
		ADMIRE	original	−0.090	−1.179 to 0.998	1	
	SAFIRE	FBP	original	−0.539	−1.627 to 0.550	0.685	
		ADMIRE	original	−0.629	−1.717 to 0.460	0.481	
	ADMIRE	FBP	original	0.090	−0.998 to 1.179	1	
		SAFIRE	original	0.629	−0.460 to 1.717	0.481	
GM	FBP	SAFIRE	90%	−1.386	−2.286 to −0.486	0.001	0.366
		ADMIRE	90%	−0.074	−0.975 to 0.826	1	
	SAFIRE	FBP	90%	1.386	0.486 to 2.286	0.001	
		ADMIRE	90%	1.312	0.412 to 2.212	0.002	
	ADMIRE	FBP	90%	0.074	−0.826 to 0.975	1	
		SAFIRE	90%	−1.312	−2.212 to −0.412	0.002	
WM	FBP	SAFIRE	90%	0.491	−0.594 to 1.576	0.810	0.442
		ADMIRE	90%	−0.106	−1.191 to 0.979	1	
	SAFIRE	FBP	90%	−0.491	−1.576 to 0.594	0.810	
		ADMIRE	90%	−0.597	−1.682 to 0.488	0.543	
	ADMIRE	FBP	90%	0.106	−0.979 to 1.191	1	
		SAFIRE	90 %	0.597	−0.488 to 1.682	0.543	
GM	FBP	SAFIRE	70%	−1.361	−2.259 to −0.463	0.001	0.366
		ADMIRE	70%	−0.027	−0.926 to 0.871	1	
	SAFIRE	FBP	70%	1.361	0.463 to 2.259	0.001	
		ADMIRE	70%	1.334	0.435 to 2.232	0.002	
	ADMIRE	FBP	70%	0.027	−0.871 to 0.926	1	
		SAFIRE	70%	−1.334	−2.232 to −0.435	0.002	
WM	FBP	SAFIRE	70%	0.482	−0.597 to 1.562	0.829	0.439
		ADMIRE	70%	−0.092	−1.172 to 0.987	1	
	SAFIRE	FBP	70%	−0.482	−1.562 to 0.597	0.829	
		ADMIRE	70%	−0.575	−1.654 to 0.505	0.587	
	ADMIRE	FBP	70%	0.092	−0.987 to 1.172	1	
		SAFIRE	70%	0.575	−0.505 to 1.654	0.587	

ROI = region of interest; SEM = standard error of mean; GM = gray matter; WM = white matter; FBP = filtered back projection; SAFIRE = sinogram-affirmed iterative reconstruction; ADMIRE = advanced modeled iterative reconstruction.

## Data Availability

In order to safeguard the confidentiality of the participants, the data pertaining to this study are currently withheld from public access. The data can be shared upon rea-sonable request.

## Supplementary Materials

The following supporting information can be downloaded at https://www.mdpi.com/article/10.3390/diagnostics15121541/s1, Figure S1: Example image of region of interest (ROI) placement in the supratentorial frontal periventricular white matter and gray matter (thalamus) locations in a mid-thalamic slice for filtered back projection (FBP), sinogram-affirmed iterative reconstruction (SAFIRE), and advanced modeled iterative reconstruction (ADMIRE) at the original dose; Figure S2: Simulated dose at 90% using ReconCT in filtered back projection (FBP), in sinogram-affirmed iterative reconstruction (SAFIRE), and in advanced modeled iterative reconstruction (ADMIRE) of non-enhanced head computed tomography (NECT). At the centrum semiovale, FBP and ADMIRE show a similar image texture with different noise intensity, while SAFIRE differs in image texture and shows multiple hyperdense spots in the white matter (orange arrows).

## Abbreviations

The following abbreviations are used in this manuscript:

ADMIRE	Advanced modeled iterative reconstruction
ALARA	As low as reasonably achievable
CNR	Contrast-to-noise ratio
CT	Computed tomography
CTDI	Computed tomography dose index
DLP	Dose length product
FBP	Filtered back projection
IR	Iterative reconstruction
NECT	Non-enhanced head CT
NPS	Noise power spectrum
SAFIRE	Sinogram-affirmed iterative reconstruction
