# Supplementary figures and images for: Comparison of ADMIRE, SAFIRE, and Filtered Back Projection in Standard and Low-Dose Non-Enhanced Head CT
Source: Diagnostics (Basel). 2025 Jun 17;15(12):1541. doi: 10.3390/diagnostics15121541 (PMC12192471; doi:10.3390/diagnostics15121541)

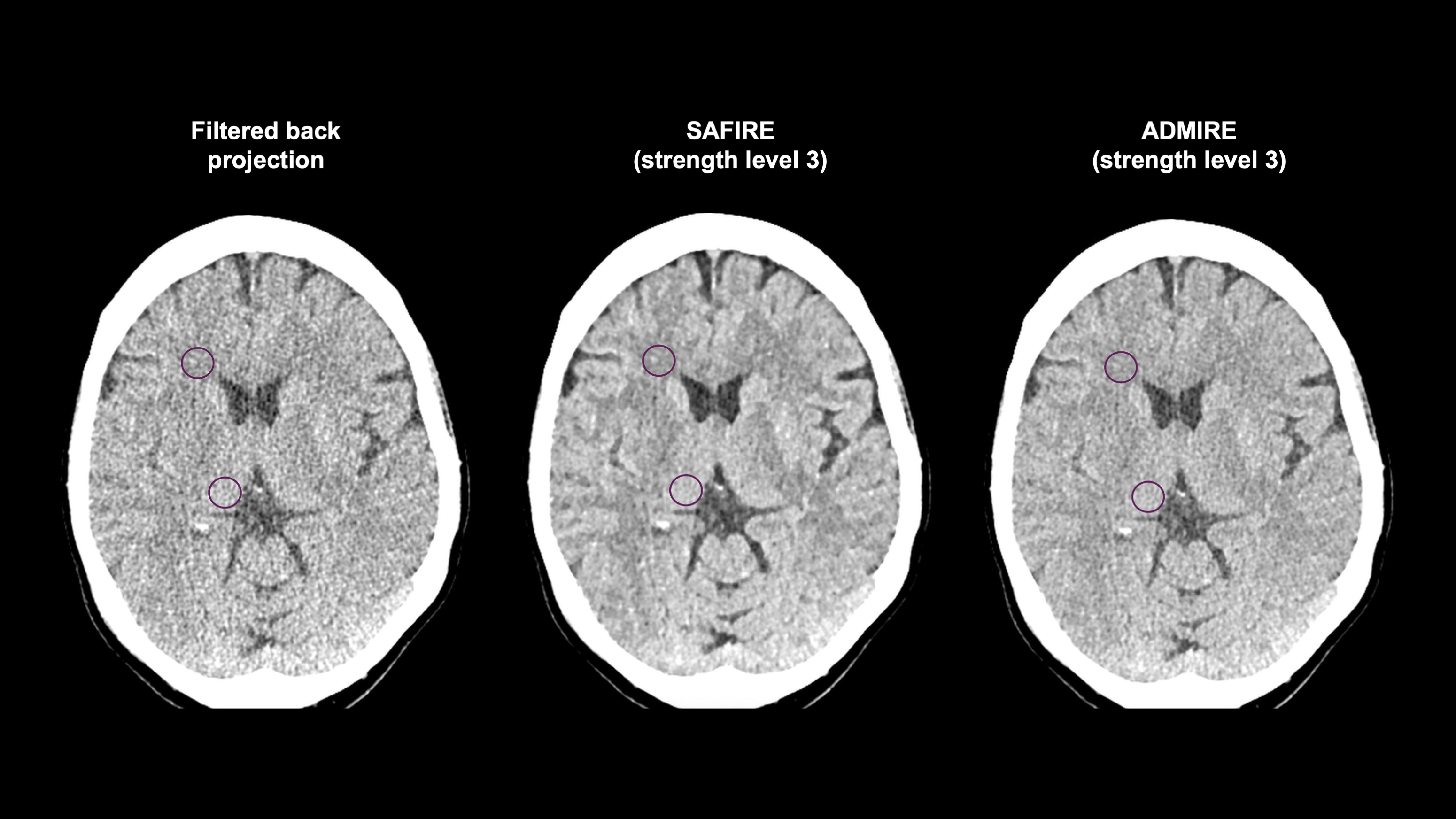

Supplement: Supplementary file 1 [file diagnostics-15-01541-s001.zip › Supplementary Figure S1.tiff]

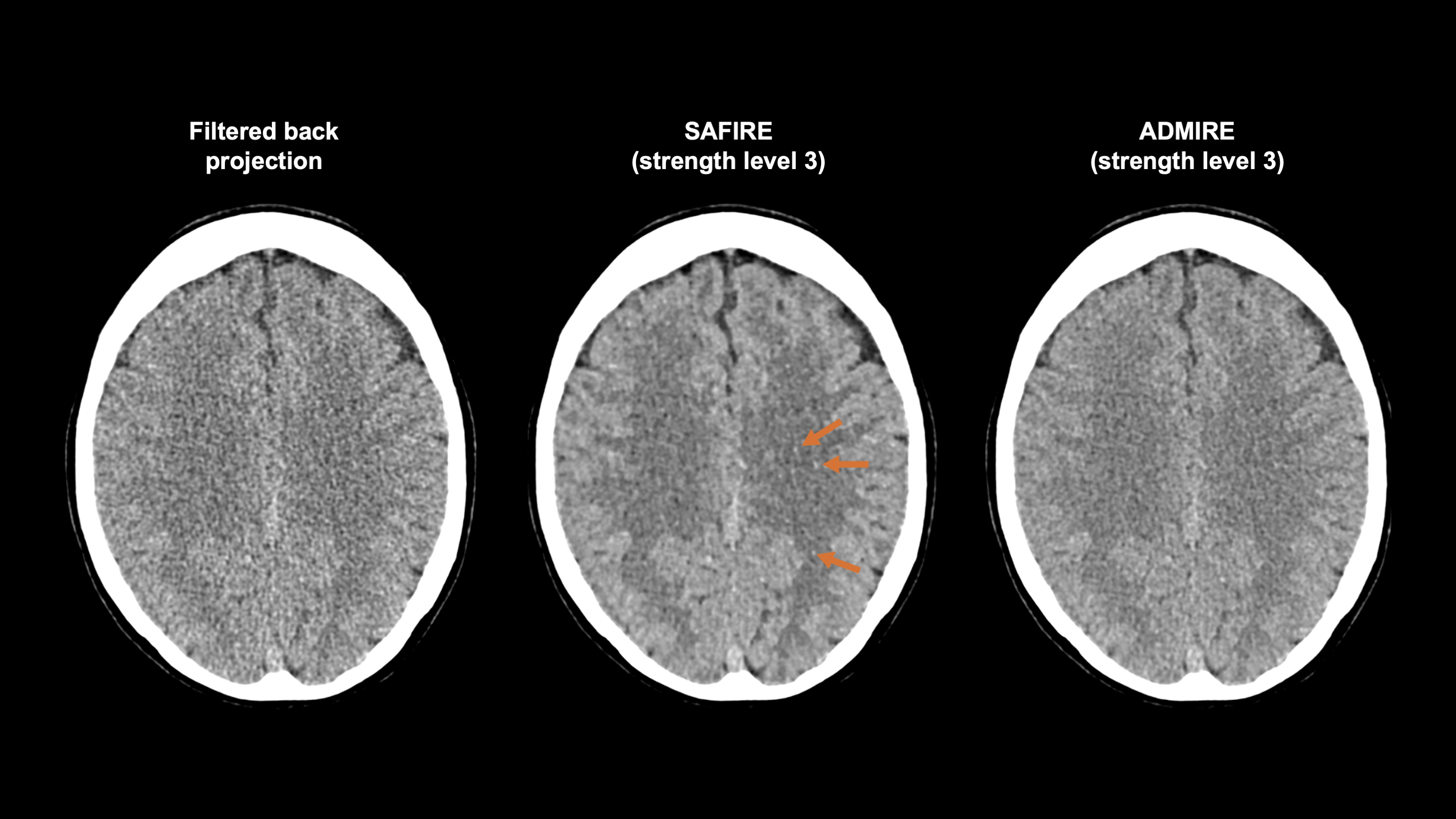

Supplement: Supplementary file 1 [file diagnostics-15-01541-s001.zip › Supplementary Figure S2.tiff]
